# The Age-adjusted Charlson Comorbidity Index predicts post-operative delirium in the elderly following thoracic and abdominal surgery: A prospective observational cohort study

**DOI:** 10.3389/fnagi.2022.979119

**Published:** 2022-08-17

**Authors:** Jing Liu, Jianli Li, Jinhua He, Huanhuan Zhang, Meinv Liu, Junfang Rong

**Affiliations:** ^1^Department of Anesthesiology, Hebei General Hospital, Shijiazhuang, China; ^2^Graduate Faculty, Hebei North University, Zhangjiakou, China

**Keywords:** elderly, Age-adjusted Charlson Comorbidity Index, thoracic and abdominal surgery, observational study, post-operative delirium

## Abstract

**Background:**

Post-operative delirium (POD) presents as a serious neuropsychiatric syndrome in the elderly undergoing thoracic and abdominal surgery, which is mostly associated with poor prognosis. The Age-adjusted Charlson Comorbidity Index (ACCI) has been widely recognized as an independently predictive factor for overall survival rate and mortality in various surgeries. However, no studies demonstrated the potential relationship between ACCI and POD. The current study was to explore the correlation between ACCI and POD, and determine the predictive effect of ACCI on POD in the elderly after thoracic and abdominal surgery.

**Materials and methods:**

Total 184 patients (≥60 years) who underwent thoracic and abdominal surgery from 2021.10 to 2022.5 were enrolled in this prospective observational cohort study. ACCI was calculated by weighting comorbidities and age. POD was diagnosed using Confusion Assessment Method (CAM) twice a day in the first 3 days after surgery. The Visual Analog Scale (VAS) was applied to measure pre-operative and post-operative pain at rest and in motion. All demographic and perioperative data were compared in patients with POD and without POD. ACCI and other variables were analyzed by univariate and multivariate logistic regression analysis. The characteristic curve of receiver operating characteristic (ROC) was used to further evaluate the accuracy of ACCI to predict POD.

**Results:**

Post-operative delirium was diagnosed in 36 of 184 patients included in our study. The prevalence of POD in the elderly after thoracic and abdominal surgery was 19.6%. The outcomes by multivariate regression analysis showed the independent risk factors for POD were ACCI (OR: 1.834; 95%CI: 1.434–2.344; *P* < 0.001), pre-operative Mini-Mental State Examination (MMSE) scores (OR: 0.873; 95%CI: 0.767–0.994; *P* = 0.040), serum albumin (OR: 0.909; 95%CI: 0.826–1.000; *P* = 0.049) and pain scores in the post-operative third day (OR: 2.013; 95%CI: 1.459–2.778; *P* < 0.001). ACCI can predict POD more accurately with the largest area under curve (AUC) of 0.794 and sensitivity of 0.861, respectively.

**Conclusion:**

Age-adjusted Charlson Comorbidity Index, pre-operative MMSE scores, serum albumin and post-operative pain were independently associated with POD in geriatric patients following thoracic and abdominal surgery. Moreover, ACCI may become an accurate indicator to predict POD early.

## Introduction

With the aging of population, the proportion of surgeries in the elderly is increasing. It is reported that thoracic and abdominal surgery accounted for 54.8% of all surgeries among the elderly in China (Han et al., 2019). The incidences of post-operative complications in elderly patients after thoracic and abdominal surgery range from 12 to 47% and from 13 to 39%, respectively (Revenig et al., 2015; Mosquera et al., 2016). Post-operative delirium (POD), a common complication in the elderly after thoracic and abdominal surgery, exerts an acute and transient neurological disorder, mainly characterized by inattention and cognitive function decline within 1 week after surgery (Robinson et al., 2009). It is estimated that 11.1–45.6% of elderly patients can develop POD (Ho et al., 2021). In addition, the risk for POD is increasing with the aging of the population. POD can lead to various adverse consequences, such as prolonged hospitalization, higher economic costs, and an increased risk for Alzheimer’s disease (Kinchin et al., 2021; Richardson et al., 2021). Moreover, it may be even strongly associated with high mortality and morbidity (Aung Thein et al., 2020). Therefore, it is crucial to prevent POD for improving the long-term prognosis and the life quality of patients. POD is multifactorial and complex, depending on the interaction between predisposing and precipitating factors (Janssen et al., 2019; Seiler et al., 2020). As a previous meta-analysis reported, some potentially related risk factors can induce POD, such as advanced age, comorbidities, and others (Rong et al., 2021). Since 30–40% of the onset of delirium can be prevented (Ishibashi et al., 2022), it might play a prominent role in reducing POD by early identifying associated risk factors.

Charlson Comorbidity Index (CCI) was firstly proposed by Charlson et al. (1987), which has become an indicator to estimate mortality risk owing to comorbidity (Charlson et al., 2022). A meta-analysis has demonstrated that CCI ≥ 2 was independently associated with the development of POD (Mevorach et al., 2022). After adjusting age as a correction variable, Age-adjusted Charlson Comorbidity Index (ACCI) is regarded as a new index to evaluate prognosis, which is calculated ultimately by integrating age and all underlying diseases, namely, cerebrovascular disease, liver or kidney disease, and heart disease, etc., and a higher ACCI can lead to worse survival rate and more mortality (Aoyama et al., 2020). Currently, ACCI is applied to standardize the evaluation of surgical patients and to predict the post-operative mortality of patients undergoing surgery (Asano et al., 2017; González Quevedo et al., 2017). Moreover, ACCI played a remarkable role in predicting post-surgical complications such as arrhythmia, delirium, stroke, and other diseases in the orthopedic surgery (Marya et al., 2016; Amit and Marya, 2022), and the incidence and severity of post-operative complications were higher in patients with high ACCI score than those with low ACCI score (Nagata et al., 2021). Nonetheless, the direct relationship between ACCI and POD in thoracic and abdominal surgery remains obscure to date.

Given this context, we aimed to analyze ACCI and other risk factors associated with POD and determine the predictive value of ACCI on POD in the elderly after thoracic and abdominal surgery, to provide guidance for clinical management of patients.

## Materials and methods

### Study population

This prospective observational cohort study was approved by the Medical Ethics Committee of Hebei General Hospital. Elderly patients aged ≥60 years who scheduled for thoracic and abdominal surgery were screened for eligibility. The study included the participants meeting the eligibility criteria in Hebei General Hospital from October, 2021 to May, 2022. The inclusion criteria were as follows: regardless of gender and nationality, American Society of Anesthesiologists (ASA) grade II ∼ III, operation time ≥1 h, surgical procedures under general anesthesia including thoracic, gastrointestinal, urinary, hepatobiliary surgery. Patients who developed delirium before surgery, refused to participate, lacked of cooperation or communication abilities, were unable to read Chinese before surgery and entered intensive care unit (ICU) after surgery were excluded.

### Data collection

#### Demographic and clinical characteristics

Demographic data [age, ASA grade, and Body Mass Index (BMI), etc.,] and comorbidities (hypertension, cardiac arrhythmia, and coronary disease) were recorded in a medical chart. Clinical data obtained from the electronic anesthesia record included operation and anesthesia time, surgical types, drugs usage (remifentanil and sufentanil), and others. Mini-Mental State Examination (MMSE) was adopted to assess the pre-operative cognitive condition, and MMSE score of less than 27 indicated cognitive impairment (Segernäs et al., 2022). Anxiety or depression was diagnosed by Hospital Anxiety and Depression Scale (HADS) (Pais-Ribeiro et al., 2018). Pre-operative and post-operative pain was frequently described by Visual Analogue Scale (VAS) (da Costa et al., 2021).

#### Pre-operative laboratory indicators

Laboratory data included neutrophils, hemoglobin, serum albumin, D-dimer, prognostic nutrition index (PNI), and albumin to fibrinogen ratio (AFR), etc. AFR was calculated as serum albumin divided by fibrinogen. PNI was calculated by the following formula: [10 × serum albumin value (g/dl)] + [0.005 × total lymphocyte count in the peripheral blood (per mm^3^)] (Cadwell et al., 2020).

All data were acquired independently from the medical records by two researchers, which were regarded as potential variables to result in POD.

#### The Age-adjusted Charlson Comorbidity Index

Evaluation and definition of comorbidities were performed prior to thoracic and abdominal surgery. The CCI score included 19 different medical conditions, with a score range of 1–6 for each comorbidity to sum an index score. Each decade over the age of 40 years was assigned a comorbidity score of 1. ACCI was calculated by adding the CCI score and age, where a higher score indicated a poorer physical condition (Aoyama et al., 2020). Since all patients were 60 years old or over, ACCI score was not less than 2 points (Supplementary Table 1).

#### Post-operative delirium assessment and determination

Confusion Assessment Method (CAM) was used to assess POD twice a day (08:00–10:00 a.m. and 18:00–20:00 p.m.) during the post-operative first 3 days by a trained anesthesiologist who was unaware of this study. All subjects were finally divided into POD group and non-POD group according to the diagnostic criterion, based on observations in four aspects, including: (1) changes in level of consciousness, (2) an acute fluctuation in mental status, (3) disordered thinking, (4) inattention. Delirium was defined as the presence of (1) and (2), accompanied by (3) or (4) or both (González et al., 2004). At the same time, patients discharged within 3 days can be followed up by telephone.

### Statistical analyses

In our study, at least 6–10 patients per independent variable events are necessary to adequately produce estimates of effect with binary regression models (Peduzzi et al., 1996). Based on the reported incidence of POD after major abdominal surgery of approximately 17.8% (Li et al., 2021), a least sample size of 141 individuals will allow 4 variables to be assessed in the regression model. Data analyses were conducted by IBM SPSS statistics software version 25.0 (SPSS Inc., Chicago, IL, United States). Quantitative data were described as mean and standard deviation ($\bar{x}$ ± *s*) or as median and interquartile ranges [M (IQR)], depending on the normality of the variables checked by Shapiro–Wilk (SW) test. For continuous variables, differences in both groups can be compared either by independent sample *t*-test or Mann–Whitney *U* test. On the other hand, categorical variables were represented as number (*n*) or rate (%), which can be tested by chi-square test or Fisher test. *P* < 0.05 was identified as statistically significant. Using tolerance (Tol) and variance inflation factor (VIF) examined multicollinearity among variables. The arguments with *P*-value < 0.1 by univariate regression analysis were performed a forward stepwise multivariate logistic regression analysis, thus controlling the confounding bias and screening out related risk factors for POD. The odds ratio (OR) with 95% confidence interval (CI) and the *P*-values were used to express the effects of related variables. Moreover, we implemented Hosmer and Lemeshow goodness-of-fit test to verify the model fitness for the logistic regression. In addition, the characteristic curve of receiver operating characteristic (ROC) was applied as a descriptive tool to further evaluate the accuracy of ACCI in predicting POD in terms of the area under curve (AUC).

## Results

### Comparison of patients characteristics

**FIGURE 1:**
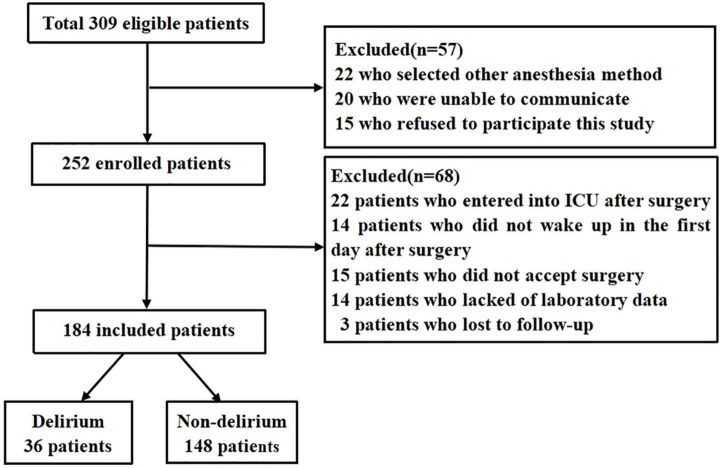
Flow chart of study population.

Initially, total 309 elderly patients were included in this study, of whom 125 patients were excluded because of poor communication, data loss, refusal surgery, and other reasons, and 184 patients were enrolled to analyze ultimately (Figure 1). The incidence of POD in the elderly following thoracic and abdominal surgery was 19.6%. All demographic and clinical data were displayed in Table 1, where the median (interquartile ranges) age was 68 (64–72) years and males were 102 cases (54.4%). Among demographic data, there were statistic differences in age (*P* = 0.004), educational level (*P* = 0.022), ASA grade (*P* = 0.004), BMI (*P* = 0.028), and pre-operative MMSE scores (*P* = 0.010) between POD group and non-POD group. The level of ACCI [7 (6–8) vs. 5 (4–6), *P* < 0.001] was higher in patients with POD, in comparison with that without POD. Pain scores in the first 3 days after surgery had significant differences in two groups (all *P* < 0.05).

**TABLE 1:** Comparison of demographic and clinical data between two groups.

Variables	Total (*N* = 184)	Delirium (*n* = 36)	Non-delirium (*n* = 148)	*P*-value
Age (years)	68 (64–72)	70.5 (67–75)	67 (64–72)	0.004
Gender (male)	102 (55.4)	20 (55.6)	82 (55.4)	0.987
Educational level (*n*%)				**0.022**
Low degree (Illiteracy and primary school)	72 (39.1)	12 (33.3)	60 (40.5)	–
Medium degree (Middle and senior school)	90 (48.9)	23 (63.9)	67 (45.3)	–
High degree (College and above)	22 (12.0)	1 (2.8)	21 (14.2)	–
BMI (kg/m^2^)	25.3 ± 3.2	24.2 ± 2.6	25.5 ± 3.3	**0.028**
ASA grade (*n*%)				**0.004**
II	86 (46.7)	9 (25.0)	77 (52.0)	–
III	98 (53.3)	27 (75.0)	71 (48.0)	–
Smoking history (*n*%)	44 (23.9)	13 (36.1)	31 (20.9)	0.056
Alcohol consumption (*n*%)	21 (11.4)	6 (16.7)	15 (10.1)	0.269
Pre-operative MMSE scores (0–30)	27 (24–28)	25 (21.3–28)	27 (25–28)	**0.010**
Cognitive impairment (*n*%)	90 (48.9)	69 (46.6)	21 (58.3)	0.207
Anxiety (*n*%)	31 (16.8)	6 (16.7)	25 (16.9)	0.974
Depression (*n*%)	4 (11.1)	2 (5.6)	2 (1.4)	0.361
ACCI	5 (4–7)	7 (6–8)	5 (4–6)	<**0.001**
Comorbidities				
Hypertension (*n*%)	89 (48.4)	19 (52.8)	70 (47.3)	0.555
Cardiac arrhythmia (*n*%)	29 (15.8)	6 (16.7)	23 (15.5)	0.701
Coronary heart disease (*n*%)	24 (13.0)	4 (11.1)	20 (13.5)	0.914
Pre-operative pain scores	0 (0–0)	0 (0–0)	0 (0–0)	0.185
Operation time (min)	152.5 (100–217.5)	175 (101.2–233.8)	145 (98.5–200)	0.147
Anesthesia time (min)	195 (145–263.8)	234 (146.3–278.8)	190 (141.3–253.8)	0.065
Surgical methods (endoscopic) (*n*%)	152 (82.6)	30 (83.3)	122 (82.4)	0.898
Type of surgery (*n*%)				0.422
Thoracic	95 (51.6)	23 (63.9)	72 (48.6)	–
Gastrointestinal	44 (23.9)	6 (16.7)	38 (25.7)	–
Urinary	34 (18.5)	5 (13.9)	29 (19.6)	–
Hepatobiliary	11 (6.0)	2 (5.5)	9 (6.1)	–
Dosage of opioids				
Remifentanil (mg)	1.5 (1.1–2.4)	1.8 (1.1–2.9)	1.5 (1–2.4)	0.185
Sufentanil (μg)	25 (20–30)	25 (20–30)	25 (20–30)	0.507
Norepinephrine usage (mg)	0 (0–0.1)	0 (0–0.3)	0 (0–0.1)	0.653
Estimated blood loss volume (ml)	121.4 (72.5–229.6)	151.4 (99.4–231.4)	113.1 (53.6–226.7)	0.103
Infusion of blood products				
Red blood cells (U)	0 (0–0)	0 (0–0)	0 (0–0)	0.389
Plasma (ml)	0 (0–0)	0 (0–0)	0 (0–0)	0.249
Minimum body temperature (°C)	36.3 (36.1–36.4)	36.3 (36.1–36.4)	36.3 (36.1–36.4)	0.942
Pain scores within the first 3 days post-operatively (0–10)				
The first day post-operatively	5 (3–6)	6 (4–6.8)	4.5 (3–6)	**0.005**
The second day post-operatively	4 (3–5)	5 (4–6)	3 (2–5)	<**0.001**
The third day post-operatively	3 (2–4)	4 (3–5.8)	3 (2–4)	<**0.001**
Use of post-operative analgesic pump (*n*%)	129 (70.1)	24 (66.7)	105 (70.9)	0.615
Total times of analgesics used within the first 3 days post-operatively	1 (0–1)	1 (1–1)	1 (0–1)	0.051
Total times of analgesics used in the first day post-operatively	1 (0–1)	1 (1–1)	1 (0–1)	0.083
Total times of analgesics used in the second day post-operatively	0 (0–0)	0 (0–0)	0 (0–0)	0.193
Total times of analgesics used in the third day post-operatively	0 (0–0)	0 (0–0)	0 (0–0)	0.244
Post-surgical stay (days)	7 (5–9)	7.5 (5–10)	7 (5–9)	0.333
Length of hospital stay (days)	12 (8–16.8)	13.5 (9–19.5)	12 (8–15)	0.333

Bold values indicated statistical significances.

Abbreviation: BMI, Body Mass Index; ASA, American Society of Anesthesiologists; MMSE, Mini-Mental State Examination; ACCI, Age-adjusted Charlson Comorbidity Index.

### Comparison of pre-operative laboratory relevant indicators

As indicated in Table 2, subjects with POD had lower levels of serum albumin (36.0 ± 5.5 vs. 38.0 ± 4.8, *P* = 0.030), AFR [10.5 (8.6–13.6) vs. 12.7 (9.9–14.9), *P* = 0.026] and total cholesterol [4.3 (3.7–4.9) vs. 4.8 (3.9–5.5), *P* = 0.037], compared to those without POD. Patients with a higher level of D-dimer had an increased risk to develop POD (*P* = 0.009).

**TABLE 2:** Pre-operative laboratory variables in older patients with or without post-operative delirium (POD).

Variables	Total (*N* = 184)	Delirium (*n* = 36)	Non-delirium (*n* = 148)	*P*-value
Neutrophil count (×10^9^/L)	4.4 (3–7)	4.9 (3.2–9)	4.3 (3–6.8)	0.187
Platelets count (×10^9^/L)	213.5 (170.8–255.5)	226.5 (161–252.8)	212 (170.8–259.5)	0.859
Total lymphocyte count (×10^9^/L)	1.3 (1–1.9)	1.5 (1–2.3)	1.3 (1–1.8)	0.249
White blood cell count (×10^9^/L)	6.6 (5.3–8.6)	7.6 (6.1–10.1)	6.4 (5.1–8.4)	0.062
Hemoglobin (g/L)	126.2 ± 16.1	121.8 ± 13.7	127.3 ± 16.4	0.063
Serum albumin (g/L)	37.6 ± 5.0	36.0 ± 5.5	38.0 ± 4.8	**0.030**
Creatinine (μmol/L)	67.3 (56.6–77.9)	71.5 (59.9–83.2)	66.1 (55.6–77.7)	0.064
Blood type (*n*%)				0.769
A	49 (26.6)	9 (25.0)	40 (27.0)	–
B	65 (35.3)	12 (33.3)	53 (35.8)	–
AB	13 (7.1)	4 (11.1)	9 (6.1)	–
O	57 (31.0)	11 (30.6)	46 (31.1)	–
Fibrinogen (g/L)	3.1 (2.7–3.8)	3.2 (2.7–4.1)	3.1 (2.6–3.7)	0.248
CK-MB (U/L)	15.6 (13.1–17.9)	15.3 (13–17.3)	15.7 (13.2–17.9)	0.455
AST/ALT	1.3 (1–1.7)	1.4 (1–1.8)	1.3 (1–1.7)	0.636
Uric acid (μmol/L)	289.7 ± 78.4	284.0 ± 63.7	291.1 ± 81.7	0.629
D-dimer (mg/L)	0.5 (0.3–0.9)	0.7 (0.5–1.3)	0.5 (0.3–0.9)	**0.009**
AFR	12.4 (9.6–14.6)	10.5 (8.6–13.6)	12.7 (9.9–14.9)	**0.026**
PNI	44.4 (40.4–49.3)	43.4 (38.6–50.2)	44.6 (40.9–49.9)	0.352
BUN (mmol/L)	4.9 (4–5.8)	5.3 (4–6.3)	4.8 (3.9–5.8)	0.300
Calcium (mmol/L)	2.2 (2.1, 2.3)	2.1 (2.0, 2.3)	2.2 (2.1, 2.3)	0.093
Sodium (mmol/L)	140 (138–141)	140 (138–141.8)	140 (138–141)	0.796
Total cholesterol (mmol/L)	4.7 (3.9–5.5)	4.3 (3.7–4.9)	4.8 (3.9–5.5)	**0.037**

Bold values indicated statistical significances.

Abbreviation: CK-MB, Creatine kinase-MB; AST/ALT, Aspartate transaminase/Alanine aminotransferase; AFR, Albumin to Fibrinogen Ratio; PNI, Prognostic Nutrition Index; BUN, Blood Urea Nitrogen.

### Multicollinearity among variables by linear analysis

Our results demonstrated that there was no severe collinearity among variables included in multivariate logistic regression analysis (all Tol >0.1, VIF <10). Details were shown in Supplementary Table 2.

### Independent risk factors for post-operative delirium by logistic regression analyses

Originally, all variables with *P* < 0.05 were performed univariate logistic regression analysis, and unadjusted outcomes that differed significantly between two groups were age, ASA grade, BMI, pre-operative MMSE scores, ACCI, serum albumin, AFR, D-dimer, total cholesterol, and pain scores within the post-operative first 3 days (all *P* < 0.1). Finally, the adjusted results by multivariate logistic regression analysis showed the independent predictors for POD were ACCI (OR: 1.834; 95%CI: 1.434–2.344; *P* < 0.001), pre-operative MMSE scores (OR: 0.873; 95%CI: 0.767–0.994; *P* = 0.040), serum albumin (OR: 0.909; 95%CI: 0.826–1.000; *P* = 0.049) and pain scores in the post-operative third day (OR: 2.013; 95%CI: 1.459–2.778; *P* < 0.001), as demonstrated in Table 3.

**TABLE 3:** Univariate and multivariate logistic regression analyses of clinical associated risk factors for post-operative delirium (POD).

Variables	Univariate			Multivariate		
	OR	95% CI	*P*-value	OR	95% CI	*P*-value
Age (years)	1.096	1.027–1.170	0.006	–	–	–
ASA grade	0.307	0.135–0.698	0.005	–	–	–
BMI (kg/m^2^)	0.874	0.774–0.987	0.030	–	–	–
Pre-operative MMSE scores	0.834	0.749–0.929	0.001	0.873	0.767–0.994	**0.040**
ACCI	1.513	1.262–1.814	<0.001	1.834	1.434–2.344	<**0.001**
Educational level (*n*%)	0.829	0.616–1.115	0.215	–	–	–
Serum albumin (g/L)	0.920	0.852–0.993	0.032	0.909	0.826–1.000	**0.049**
D-dimer (mg/L)	1.340	1.014–1.771	0.040	–	–	–
Total cholesterol (mmol/L)	0.709	0.500–1.005	0.054	–	–	–
Pain scores in the first day post-operatively	1.305	1.079–1.579	0.006	–	–	–
Pain scores in the second day post-operatively	1.451	1.178–1.787	<0.001	–	–	–
Pain scores in the third day post-operatively	1.658	1.288–2.135	<0.001	2.013	1.459–2.778	<**0.001**
AFR	0.897	0.809–0.994	0.038	–	–	–

Hosmer and Lemeshow goodness-of-fit test: χ^2^ value = 12.536, *P* = 0.129. Bold values indicated statistical significances.

Abbreviation: ASA, American Society of Anesthesiologists; MMSE, Mini-Mental State Examination; ACCI, Ageadjusted Charlson Comorbidity Index; BMI, Body Mass Index; AFR, Albumin to Fibrinogen Ratio; CI, Confidence Interval; OR, Odds Ratio.

Additionally, the predictable model fitted very well by Hosmer and Lemeshow goodness-of-fit test with a χ^2^ value 12.536 and *P*-value of 0.129.

### The predictive value of Age-adjusted Charlson Comorbidity Index for post-operative delirium by receiver operating characteristic analysis

**FIGURE 2:**
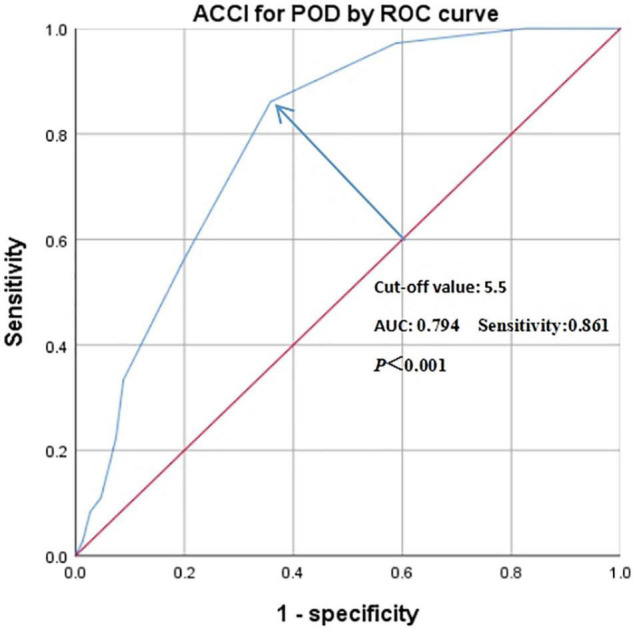
The predictive value of ACCI for POD by ROC analysis. Abbreviations: ACCI, Age-adjusted Charlson Comorbidity Index; POD, post-operative delirium; ROC, receiver operating characteristic; AUC, area under the curve.

The characteristic curve of ROC was applied to further evaluate the accuracy of all predictive factors. As presented in Table 4, ACCI had the largest AUC with 0.794 and sensitivity with 0.861, compared to others predictive risk factors (pre-operative MMSE scores AUC: 0.637 sensitivity: 0.770; serum albumin AUC: 0.609 sensitivity: 0.777; post-operative pain AUC: 0.706, sensitivity: 0.889). Also, based on ROC analysis, the optimal cut-off value of ACCI was 5.5 to predict POD (Figure 2).

**TABLE 4:** The accuracy of risk factors to predict post-operative delirium (POD) by ROC curve analysis.

Variables	Area under the curve (95% confidence interval)	Sensitivity	Specificity	Cut-off value	*P*-value
ACCI	0.794 (0.724, 0.863)	0.861	0.358	5.5	<0.001
Pre-operative MMSE scores	0.637 (0.532, 0.743)	0.770	0.556	24.5	0.011
Serum albumin (g/L)	0.609 (0.501, 0.717)	0.777	0.556	34.65	0.043
Pain scores in the third day post-operatively	0.706 (0.612, 0.800)	0.889	0.595	2.5	<0.001

Abbreviation: ACCI, Age-adjusted Charlson Comorbidity Index.

## Discussion

Post-operative delirium is a serious post-surgical complication in the elderly and contributes to various adverse effects, such as longer hospital stays, increased economic burden and decrease in life quality (Park and Lee, 2019). It has been reported that multiple related factors increased the risk of POD including advanced age, diabetes mellitus, and others (Lee et al., 2020). Early identification of related factors plays a significant role in preventing and treating POD. This prospective observational cohort study firstly explored the predictive value of ACCI on POD in geriatric patients after thoracic and abdominal surgery. Our results revealed that ACCI, pre-operative MMSE scores, serum albumin and post-operative pain were independently correlated with POD, and ACCI was regarded as a predictor for POD.

In the present study, the prevalence of POD in the elderly undergoing thoracic and abdominal surgery was 19.6%. Similar to our finding, an observational retrospective cohort study analyzed 1,055 cases of elderly patients undergoing major abdominal surgery and observed that 17.8% of patients developed POD (Li et al., 2021). Additionally, a previous study showed that POD occurred in 22.4% of elderly patients after esophagectomy (Jung et al., 2018). However, other studies revealed the incidence of POD was a little lower than that in our study, such as the 3.3% by Ishibashi et al. (2022) and 7.3% by Ida et al. (2020), which might be related to baseline characteristics, sample size, diagnostic criteria, and different interventions in patients.

Our univariate logistic regression analysis has demonstrated that 12 related variables were potential risk factors for POD, including age, ASA grade, ACCI, and others. After adjusting related factors by multivariate logistic regression analysis, ACCI was identified as an independent predictor for POD. ACCI, as a weighting index to measure the burden of comorbidities, has become a predictive factor for post-operative complications (Amit and Marya, 2022). Numerous studies have reported that elderly cancer patients with high ACCI scores had decreased progression-free survival and overall survival (Aoyama et al., 2020; Zhou et al., 2022). Our study firstly focused on the predictive role of ACCI for POD in geriatric patients undergoing thoracic and abdominal surgery. ACCI was calculated by total points based on age and 19 medical conditions, including cerebrovascular, immune systems diseases, and others (Aoyama et al., 2020). Some reports have proved that comorbidities were independently correlated with POD, such as hypertension and dementia etc. (Pérez-Ros et al., 2019; Ramos et al., 2022). Moreover, it has been well-established that patients with advanced age were more prone to develop POD (Lee et al., 2020). Conversely, our results did not support the predictive effect of age on POD. Although univariate regression analysis indicated a statistic difference in age in two groups (*P* = 0.006), the multivariate regression analysis indicated no difference (*P* = 0.273). The possible reason for the discrepancy may be related to the small age range [68, IQR: (64–72)] and insufficient sample size in this study. While age was not an independent risk factor for POD, our results confirmed that ACCI was highly related to POD. Also, increased per one-point of ACCI scores can add 1.834-fold risk of POD (OR: 1.834; 95%CI: 1.434–2.344; *P* < 0.001), which was likely that ACCI combined the effect of both age and comorbidity and probably gived true measure of the physiologic reserve. In addition, ACCI can predict cumulative minor or major post-operative complications among geriatric patients following orthopedic surgery, including delirium and stroke in the nervous system and other systems (Marya et al., 2016; Amit and Marya, 2022). Therefore, we deemed that ACCI might have a predictive value in POD in other surgeries. Besides, the cut-off values of ACCI have been widely investigated in cancer survival (Aoyama et al., 2020; Takahara et al., 2020). However, the optimal cut-off value of ACCI to predict POD in the elderly after thoracic and abdominal surgery was hardly explored. In this study, we set the optimal intercept value of ACCI as 5.5 by ROC analysis and ACCI was regarded as a better predictive factor for POD with the largest AUC of 0.794 and sensitivity of 0.861, respectively, compared with other related variables. Nevertheless, a great deal of studies should be performed to further verify whether ACCI can better predict POD in other surgeries.

Mini-Mental State Examination has been generally accepted as a simple scale to evaluate cognitive function, including 30 lists of decline symptoms totally, and lower scores represented worse cognitive condition in patients (Segernäs et al., 2022). Our results showed that pre-operative MMSE scores in POD group were two points lower than those in non-POD group and were negatively associated with POD *via* multivariate regression analysis, which suggested that patients may have some impaired brain function pre-operatively, and thus increasing the probability to develop POD (Pettemeridou et al., 2021). In line with our study, previous studies have confirmed that patients who experienced POD had lower baseline MMSE scores before surgery (Pan et al., 2019; Humbert et al., 2021). Interestingly, there was no statistic difference in cognitive impairment assessed by MMSE between two groups in our study (*P* = 0.207), which may likely that educational attainment played a confounding effect on MMSE score. Some recent studies have suggested the educational level was positively associated with MMSE score, and the threshold of MMSE for diagnosing cognitive impairment was different among patients with different educational levels (Wu et al., 2021; Cardoso et al., 2022). Even so, pre-operative assessment of cognitive status by MMSE can contribute to early preventing POD, which deserved more attention.

Additionally, serum albumin, as an indicator representing patients’ nutritional and immune status, has been verified to be closely associated with POD in orthopedic and urological surgery (Matsuki et al., 2020; Qi et al., 2020). Moreover, a meta-analysis further evidenced that serum albumin was an independent risk factor for POD in colorectal cancer surgery (Lee and Lim, 2020). Our regression analysis result concluded that the a low level of albumin prior to surgery was relevant to an increased risk of POD, which was accordance with previous studies. Unfortunately, we did not measure the difference between pre-operative and post-operative albumin (ΔAlb), which may better explain its relationship with POD.

Our study also demonstrated that another potential risk factor that affected the onset of POD was post-operative pain. As previous studies reported, post-operative pain might exert a certain promoting influence on developing POD (Denny and Such, 2018; Ding et al., 2021). Similarly, our study showed post-operative pain had a remarkable effect on POD even though adjusting several potential factors by multiple regression analysis. Meanwhile, the risk of POD added 2.421 times when per one-point of pain scores increased (OR: 2.013; 95%CI: 1.459–2.778; *P* < 0.001). With respect to analgesic remedies, there were no statistical significance in post-operative analgesic pumps usage and analgesic drugs including flurbiprofen, ketorolac tromethamine, dezocine, pentazocine between two groups, which might be due to the fact that some patients refused to take analgesics for fear of its side effects according to surgeons’ feedback.

There are also some limitations that need to be addressed. First, since the sample size of this single-center prospective observational study is small, internal bias cannot be avoided. Second, the time of evaluating delirium is only within 3 days after surgery, which may lead to a lower incidence of POD. Finally, some other factors with an early vigilant role in POD in elderly patients, such as frailty, malnutrition and depth of anesthesia, are not fully considered in this study.

## Conclusion

Taken together, delirium was common among geriatric patients undergoing thoracic and abdominal surgery in our study. We found that ACCI, pre-operative MMSE scores, serum albumin, and post-operative pain became the independent risk factors for POD, and ACCI can better predict the development of POD. This study provides evidence supporting ACCI as a part of clinical assessments for delirium risk in elderly patients following thoracic and abdominal surgery. In order to provide evidence-based prevention strategies, clinicians should regard ACCI as an early detection to identify older patients at risk of delirium.

## Data availability statement

The original contributions presented in the study are included in the article/Supplementary material, further inquiries can be directed to the corresponding author.

## Ethics statement

The studies involving human participants were reviewed and approved by the Medical Ethics Committee of Hebei General Hospital. The patients/participants provided their written informed consent to participate in this study. Written informed consent was obtained from the individual(s) for the publication of any potentially identifiable images or data included in this article.

## Author contributions

JL, JFR, and JLL: design or idea of the study and drafting of manuscript. JL and JHH: data collection. HHZ and MNL: data monitoring and analysis. All authors contributed to revision and agreed with this manuscript.
