# Supplementary material for: The Age-adjusted Charlson Comorbidity Index predicts post-operative delirium in the elderly following thoracic and abdominal surgery: A prospective observational cohort study
Source: Front Aging Neurosci. 2022 Aug 17;14:979119. doi: 10.3389/fnagi.2022.979119 (PMC9428551; doi:10.3389/fnagi.2022.979119)
Supplement: Supplementary file 1 [file Table_1.DOCX]

**Supplement 1 Age-adjusted Charlson Comorbidity Index (ACCI)**

| Age-adjusted Charlson Comorbidity Index(ACCI) | |
| --- | --- |
| Item | points |
| One point for each of the following diagnosis  Myocardial infarction (MI)  Congestive heart-failure (CHF)  Peripheral vascular disease  Cerebrovascular disease (CVD)  Dementia  Chronic obstructive pulmonary disease (COPD)  Connective tissue disease (CTD)  Digestive ulcer disease  Diabetes (ordinary type 1 point; 2 points with other organ damage) |  |
| Two points for each of the following diagnosis  ① Moderate or severe chronic kidney disease  ② Hemiplegic paralysis  ③ Leukaemia  ④ Malignant lymphadenoma |  |
| Three points for each of the following diagnosis  ① Liver disease (mild 1 point, moderate or severe 3 points) |  |
| Six points for each of the following diagnosis  Solid tumor (No transfer 2 points, transfer 6 points)  Acquired Immune Deficiency Syndrome (Aids) |  |
| Age: add 1 point per 10 years over 40 years  <50; 1 points 50-59; 2 points 60-69; 3 points 70-79; 4 points 80-89. | |
