# Supplementary material for: The Age-adjusted Charlson Comorbidity Index predicts post-operative delirium in the elderly following thoracic and abdominal surgery: A prospective observational cohort study
Source: Front Aging Neurosci. 2022 Aug 17;14:979119. doi: 10.3389/fnagi.2022.979119 (PMC9428551; doi:10.3389/fnagi.2022.979119)
Supplement: Supplementary file 2 [file Table_2.DOCX]

**Supplement 2 Collinearity analysis of related variables included in multivariate logistic regression analysis**

| Collinear statistics | | |
| --- | --- | --- |
| Variables | Tolerance | Variance inflation factor |
| Age (years) | .795 | 1.258 |
| ASA grade | .806 | 1.240 |
| ACCI | .763 | 1.311 |
| BMI (kg/m²) | .875 | 1.143 |
| Preoperative MMSE scores | .837 | 1.195 |
| Serum albumin (g/L) | .524 | 1.908 |
| D-dimer (mg/L) | .751 | 1.331 |
| Total cholesterol (mmol/L) | .820 | 1.220 |
| Pain scores in the first day postoperatively | .297 | 3.367 |
| Pain scores in the second day postoperatively | .181 | 5.522 |
| Pain scores in the third day postoperatively | .253 | 3.960 |
| AFR | .566 | 1.767 |
| Abbreviations: BMI:Body Mass Index  MMSE: Mini-Mental State Examination  ASA: American Society of Anaesthesiologists AFR: Albumin to Fibrinogen Ratio  ACCI: Age-adjusted Charlson Comorbidity Index | | |
